# KIF18A inactivates hepatic stellate cells and alleviates liver fibrosis through the TTC3/Akt/mTOR pathway

**DOI:** 10.1007/s00018-024-05114-5

**Published:** 2024-02-19

**Authors:** Hao Zhang, Tong Xia, Zhijia Xia, Huaxin Zhou, Zhipeng Li, Wei Wang, Xiangyu Zhai, Bin Jin

**Affiliations:** 1 Organ Transplant Department, Qilu Hospital of Shandong University, Jinan, China; 2 Department of Hepatobiliary Surgery, The Second Hospital of Shandong University, Jinan, China; 3 Department of General, Visceral, and Transplant Surgery, Ludwig-Maximilians-University Munich, Munich, Germany; 4 Medical Integration and Practice Center, Shandong University, Jinan, China

**Keywords:** Liver fibrosis, HSCs, KIF18A, TTC3, AKT, mTOR pathway

## Abstract

Activation of hepatic stellate cells (HSCs) has been demonstrated to play a pivotal role in the process of liver fibrogenesis. In this study, we observed a decrease in the expression of KIF18A in fibrotic liver tissues compared to healthy liver tissues, which exhibited a negative correlation with the activation of HSCs. To elucidate the molecular mechanisms underlying the involvement of KIF18A, we performed in vitro proliferation experiments and established a CCl4-induced liver fibrosis model. Our results revealed that KIF18A knockdown enhanced HSCs proliferation and reduced HSCs apoptosis in vitro. Mouse liver fibrosis grade was evaluated with Masson’s trichrome and alpha-smooth muscle actin (α-SMA) staining. In addition, the expression of fibrosis markers Col1A1, Stat1, and Timp1 were detected. Animal experiments demonstrated that knockdown of KIF18A could promote liver fibrosis, whereas overexpression of KIF18A alleviated liver fibrosis in a CCl4-induced mouse model. Mechanistically, we found that KIF18A suppressed the AKT/mTOR pathway and exhibited direct binding to TTC3. Moreover, TTC3 was found to interact with p-AKT and could promote its ubiquitination and degradation. Our findings provide compelling evidence that KIF18A enhances the protein binding between TTC3 and p-AKT, promoting TTC3-mediated ubiquitination and degradation of p-AKT. These results refine the current understanding of the mechanisms underlying the pathogenesis of liver fibrosis and may offer new targets for treating this patient population.

**Supplementary Information:**

The online version contains supplementary material available at 10.1007/s00018-024-05114-5.

## Introduction

It is well-established that liver fibrosis results from chronic liver damage, leading to the development of fibrotic tissue and inflammatory responses [1]. During hepatic fibrosis, extracellular matrix (ECM) components accumulate in the interstitial space [2, 3]. At present, there is no clinically safe and efficient pharmacological treatment for patients with liver fibrosis [4, 5]. Accordingly, it is critical to unveil the mechanisms underlying the pathogenesis of hepatic fibrosis and identify more potent anti-fibrotic medications for treating this patient population.

Current evidence suggests that the development of fibrosis depends heavily on the activation of hepatic stellate cells (HSCs) [6]. In response to hepatic injuries, the activated HSCs adopt a myofibroblastic phenotype responsible for hepatic fibrogenesis [7, 8]. It has been established that the activation of HSCs into fibroblast-like cells is a hallmark of hepatic fibrogenesis [9, 10]. Hence, the study of HSC biology may provide new insights into the mechanism and treatment of liver fibrosis.

The kinesin-8 family, which has 45 kinesins, includes the kinesin-18 family member 18A (KIF18A) [11]. KIF18A controls chromosome congression and kinetochore motions, which have been shown to have an impact on cell proliferation in a recent study [12]. There is mounting evidence that KIF18A regulates the development of lung cancer, breast cancer, colorectal cancer, and other cancers [13–15]. Yet, it is still not entirely known how KIF18A contributes to liver fibrosis. Here, we demonstrate the downregulation of KIF18A in HSCs and liver fibrosis tissues, and identify its role in HSC activation and hepatic fibrosis induction through Akt/mTOR-signaling pathway.

As a transcription factor, Yin Yang 1 (YY1) can activate or repress gene expression [16, 17]. There is an increasing consensus suggesting that the expression of YY1 increased in many cancers and is associated with patient survival [18, 19]. Recent research indicates that cell proliferation is closely related to YY1 dosage, with YY1 knockout resulting in cytokinesis failure and cell cycle arrest [20]. Few prior studies have investigated the role of YY1 in liver fibrosis. In this research, the results of the Chromatin Immunoprecipitation (ChIP) assay confirmed the binding of YY1 to the promoter region of KIF18A upstream of the transcription start site. Furthermore, we examined the expression of YY1 in fibrotic liver tissue and investigated its role in the pathogenesis of liver fibrosis.

Overall, our findings indicate that KIF18A expression is reduced in human liver fibrosis, which promotes the activation of HSCs. We also documented that KIF18A could interact with TTC3 to regulate the ubiquitination and degradation of p-AKT. In addition, our study revealed that YY1 regulates KIF18A to deactivate HSCs through the TTC3/p-Akt/mTOR pathway. The findings of this study offer potential therapeutic targets for the treatment of liver fibrosis.

## Materials and methods

### Gene expression omnibus (GEO) data set

The mRNA expression alterations associated with the progression of human liver fibrosis were examined using the GEO data set (GSE197112) retrieved from the GEO database (https://www.ncbi.nlm.nih.gov/geo/). Gene expression patterns were analyzed in a cohort comprising four individuals with liver fibrosis and four healthy controls.

### Isolation of HSCs from livers

HSCs were isolated by in situ collagenase perfusion of the liver. HSCs were isolated from mouse livers as previously described [21]. Briefly, after washing with PBS solution, the livers were cut into small pieces. The premixed working solution was mixed and added to the liver tissues. After centrifugation and washing multiple times, the precipitates containing the HSCs were collected.

### Cell culture

The cell bank of the Chinese Academy of Sciences (Shanghai, China) provided the human HSC cells LX2. Primary HSCs and LX2 Cells were cultured in Dulbecco’s modified Eagle’s medium (DMEM, Pricella, Wuhan, China), which was supplemented with 10% fetal bovine serum, 1% penicillin, and 1% streptomycin. Cells were grown in a humidified 5% CO_2_ environment.

### Transfection

Lentivirus transfection was conducted as previously described [22]. Lentivirus vectors of KIF18A knockdown (shKIF18A), YY1 knockdown(shYY1), KIF18A(KIF18A) overexpression, and an empty lentiviral vector (shNC or Vector) were purchased from TSINGKE (Beijing TSINGKE Biotech Co., Ltd., China). Lentivirus transfection and cell line establishment viral particles were produced using 293 T cells. The transfection agent Jet PEI was used to carry out lentivirus transfection (Polyplus Transfection, San Marcos, CA). The control plasmid, Flag-KIF18A, HA-TTC3, His-UB, and MYC–AKT plasmids were created by TSINGKE. As previously mentioned, plasmid transfection was carried out using lipofectamine 3000 (Invitrogen, Carlsbad, CA, USA) [23].

### RT-qPCR

KIF18A expression in tissues was detected using qRT-PCR assays. TRIzol (Beyotime, Wuhan, China, #R0016) was used to extract total RNA. Then RNA was reversed to synthesize cDNA using a reverse transcription kit (Vazyme, Nanjing, China). BIO-RAD (Bio-Rad, US) equipment and SYBR Mix (Vazyme) were used for qRT-PCR. The primer sequences for KIF18A in human were as follows: forward primer: 5′-AAAAAGTGGTAGTTTGGGCTGA-3' and reverse primer: 5′-CTTTCAAGGGAGATGGCATTAG-3'. Human β-actin forward primer: 5′-GTGGGGCGCCCCAGGCACCAGGGC-3'; Human β-actin reverse primer: 5'-CTCCTTAATGTCACGCACGATTTC-3'. The details of PCR primers used for KIF18A, β-actin, and YY1 in human or mouse tissue are provided in Supplementary Table S1.

### Western blotting

SDS lysis buffer was used to lyse the cell samples. The lysate was loaded and analyzed by SDS–PAGE as described before [24]. Proteins were transferred to PVDF membranes (Thermo Fisher, #88,518) and blocked for an hour with 5% skim milk. Membranes were incubated with corresponding primary antibodies for 12 h at 4 °C and then incubated with secondary antibodies for 1 h at room temperature. Membranes were developed with an ECL kit (Beyotime, #P0018S) in the Tanon system (Tanon Science and Technology, Shanghai, China). Antibody details are provided in Supplementary Table S2.

### Mouse models of liver fibrosis

C57BL/6 mice (male, 8–10 weeks, 20–28 g) were included in the study. Hepatic fibrosis mice models were induced using carbon tetrachloride (CCl4). In this respect, mice received intraperitoneal injections of CCl4 in olive oil (10% CCl4) twice weekly at 5 μl/g body weight. PBS was administered to the control group mice, and after 14 weeks, the mice were sacrificed under anesthesia. Liver tissues were collected for immunohistochemistry (IHC) staining, Masson’s Trichrome histological staining, qRT-PCR, and Western blotting analyses.

After the establishment of mice liver fibrosis models, the mice were infected with adeno-associated viruses (AAVs) via tail vein injection. Delivery of AAVs carrying KIF18A (ad-KIF18A) or control AAVs (ad-Control) into mouse liver was achieved by intravenous injection via tail vein with 1 × 10^11^ AAV virus per mice in 200 μl saline (0.9% NaCl). AAVs carrying shRNAs targeting KIF18A (ad-shKIF18A) were used to target the KIF18A for knockdown through tail vein injection. Control mice were injected with control AAVs (ad-shNC). Whole livers were harvested 2 week post AAVs treatment for histological examination and mRNA and protein expression detection.

### Immunohistochemistry and Masson’s trichrome histological staining

IHC staining was conducted using COL1A1 Ready-To-Use IHC Kit (Proteintech, #KHC0205) and STAT1 Ready-To-Use IHC Kit (Proteintech, # KHC1036) according to the kit instructions. After deparaffinization and hydration, the tissue sections were sequentially treated in an antigen retrieval buffer. Primary antibody staining was performed overnight at 4 °C. These slides were then subjected to secondary antibodies for 1 h. For detection, we used the DAB Reagent kit (Vector Laboratories). For IHC staining of Timp1 and Tgfβ1, antibodies against Timp1 (Proteintech, No. 16644-1-AP) and Tgfβ1 (Proteintech, No. 21898-1-AP) were used as primary antibodies.

To visualize the deposited extracellular matrix, Masson’s trichrome staining was performed (Solarbio, Beijing, China, #G1340). Briefly, sections were soaked in Bouin’s solution overnight. The slides were then stained with Weigert’s hematoxylin for 5 min after being rinsed for 10 min under running water. The slides were then rinsed, stained with scarlet-acid fuchsin for 5 min, and then rinsed again. After that, for a total of 5 min, the slides were stained with phosphotungstic/phosphomolybdic, aniline blue, and 2% acetic acid. The slides were then polished, dried, and mounted.

### Cell proliferation assays

Using the Cell Counting Kit-8 (CCK-8, Beyotime, #C0037), cell viability was determined. LX-2 cells were grown in 96-well plates in an incubator for 24, 48, and 72 h. The cells were then exposed to around 10 µl of the CCK-8 solution and incubated at 37 °C for 2 h. Finally, a microplate reader was used to measure the absorbance at 450 nm (iMark Microplate Reader; Bio-Rad Laboratories, Inc., Hercules, CA). An EdU staining kit (Beyotime, #C0071S) was used for the process. A fluorescence microscope was used to examine the EdU-positive cells.

### Cell apoptosis

The Annexin V-FITC Apoptosis Detection Kit (Beyotime, #C1062S) was used in accordance with the manufacturer’s instructions to identify cell apoptosis [22]. In short, a total of 5 × 10^5^ cells were harvested and underwent two cold PBS washes. 100 µl of cell suspension was mixed with 10 µl of V-FITC and 10 µl of propidium iodide, and then incubated for 15 min. The cells were immediately examined by flow cytometry after staining (Beckman CytoFlex, USA).

### Immunoprecipitation (IP) and ubiquitination assays

Immunoprecipitation assays to detect ubiquitinated proteins were performed as previously documented [25]. Antibody against p-AKT for IP assays was purchased from Cell-Signaling Technology (Danvers, MA, USA). Briefly, LX-2 cells were transfected with plasmids encoding His-ubiquitin and other proteins. After 48 h, 10 nM MG132 and SC79 were added, and cells were incubated for 8 h. The LX-2 cells were then collected, and p-AKT or TTC3 protein was immunoprecipitated as the primary antibody. Then TTC3 or p-AKT was detected by Western blot. For ubiquitination assay, after IP using A/G beads (Life Technologies) and antibody against p-AKT, the eluted protein was detected by Western blot analysis using an anti-His-ubiquitin antibody. The specifications of the antibody utilized in IP can be found in Supplementary Table S2.

### Chromatin immunoprecipitation assay

The ChIP assay was carried out in accordance with prior descriptions [26]. The cells were briefly treated with formaldehyde. After that, chromatin immunoprecipitation lysis buffer was used to lyse the cells. After being split into IgG and IP groups, the samples underwent immunoprecipitation. qRT-PCR was used to examine the ChIP DNA. The primer sequences utilized for the ChIP experiment can be located in Supplementary Table S3.

### Statistical analyses

All immunohistochemical analyses were performed using ImageJ software. All statistical analyses were performed with GraphPad Prism 8.0 (GraphPad Prism version; GraphPad Software, https://www.graphpad.com/) or SPSS 21.0 (SPSS Standard, Chicago, IL, USA). The Chi-square test and *t* test were used for parameter analysis. All experiments were repeated three times unless specified otherwise, and *P* values < 0.05 were statistically significant. *P < 0.05, **P < 0.01, and ***P < 0.001.

## Results

### KIF18A expression is downregulated in liver fibrosis

**Fig. 1:**
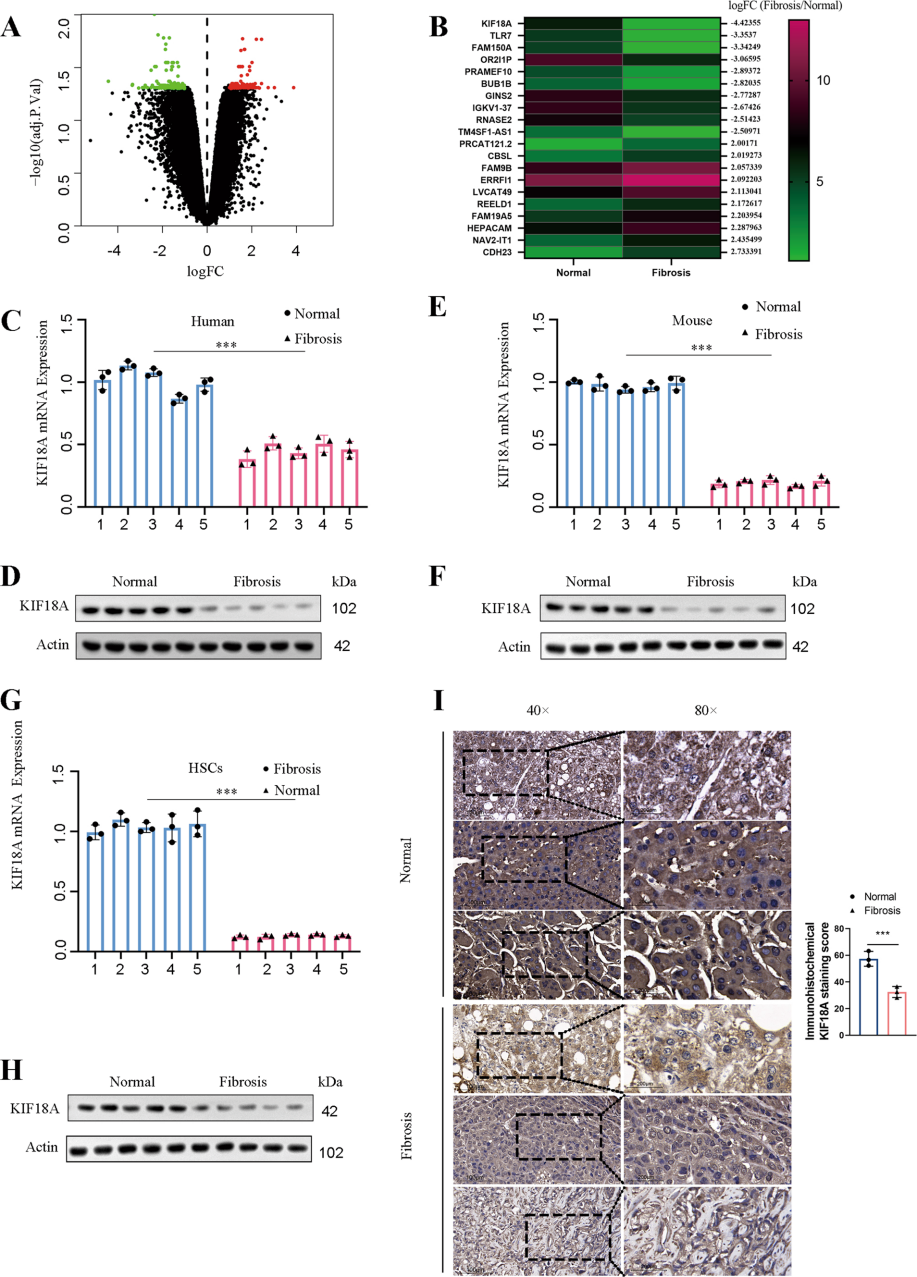
KIF18A expression is downregulated in liver fibrosis.** A** Volcano map of differentially expressed genes in GEO: GSE197112. **B** Heat map displaying the expression levels of the top 20 genes with significant differential expression, as determined by the analysis of differential gene expression in GSE197112. **C** KIF18A mRNA expression in human fibrotic tissues and healthy liver tissues. **D** Expression of KIF18A protein was detected by western blot analysis in human fibrotic liver tissues and healthy liver tissues. **E** KIF18A mRNA expression decreased in the liver fibrosis mouse model. **F** KIF18A protein expression decreased in the liver fibrosis mouse model. **G** KIF18A mRNA expression decreased in HSCs isolated from the liver fibrosis mouse model. **H** KIF18A protein expression decreased in HSCs isolated from the liver fibrosis mouse model. **I** Immunohistochemical staining of fibrotic liver tissue and normal liver tissue obtained from clinical sources demonstrated a decrease in KIF18A expression in fibrotic liver tissue. (immunohistochemical staining scale bar, 100 μm). *P < 0.05, **P < 0.01, and ***P < 0.001

We obtained transcriptomic sequencing data of liver fibrosis tissue and normal liver tissues from the GEO database (GSE197112). Differential analysis results revealed 309 differentially expressed genes with a *p* value < 0.05 (Fig. 1A). Notably, KIF18A expression displayed the most substantial downregulation (Fig. 1B). Human clinical samples of liver fibrosis tissues and normal liver tissues were collected from Qilu hospital of Shandong university. We found that KIF18A significantly decreased in the human liver fibrosis tissues by qRT-PCR detection (Fig. 1C). Consistently, western blot showed that KIF18A protein expression was low in liver fibrosis (Fig. 1D). Subsequently, we evaluated the expression level of KIF18A in a mouse model of liver fibrosis to ascertain whether it was downregulated in a manner consistent with clinical samples. To establish a mouse model of liver fibrosis, CCl4 was injected, and the mice were sacrificed to obtain liver samples. The results of qRT-PCR and Western blot showed that both mRNA and protein expression levels of KIF18A were lower in the liver samples of fibrosis models than in the control group mice (Fig. 1E, F). Considering the crucial role played by HSCs in the process of liver fibrosis, it is imperative to further ascertain the significant alterations in the expression of KIF18A within activated HSCs in fibrotic livers. We isolated primary HSCs from the livers of both fibrosis and control mice to examine the expression of KIF18A. Our findings demonstrated a downregulation of KIF18A mRNA and protein expression in activated HSCs of mice with CCl4-induced fibrosis (Fig. 1G, H). To elucidate the protein expression alterations of KIF18A in hepatic fibrotic tissues, we employed immunohistochemical staining. Immunohistochemistry staining showed that KIF18A expression was downregulated in human fibrotic liver tissues (Fig. 1I). Collectively, these results indicate that KIF18A expression is decreased in liver fibrosis tissues and HSCs.

### KIF18A knockdown promotes the proliferation and decreases apoptosis of HSCs

**Fig. 2:**
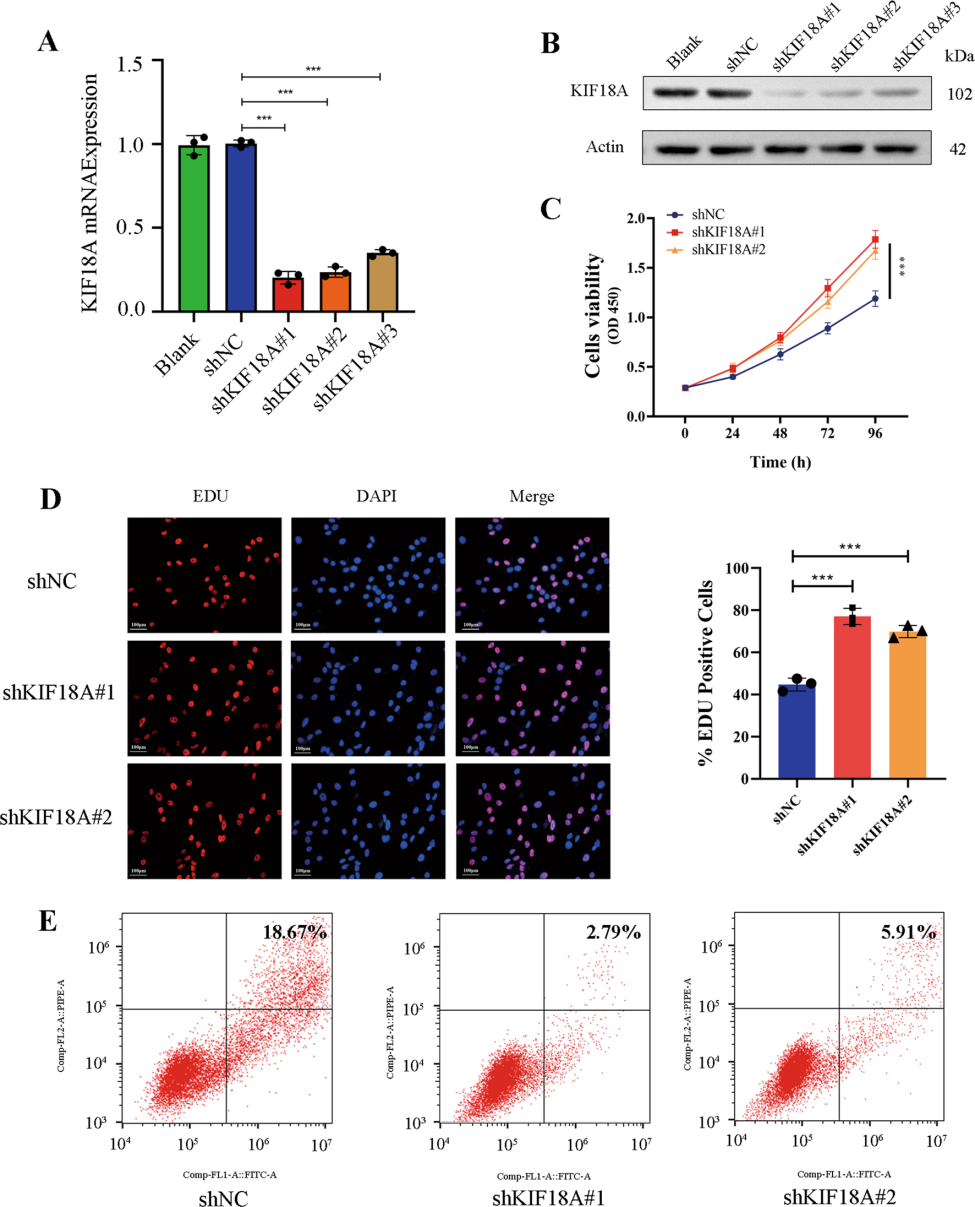
KIF18A knockdown promotes the proliferation and decreases apoptosis of HSCs. **A** Three different lentiviruses containing distinct sequences targeting KIF18A were transfected into LX-2 cells for knockdown of KIF18A expression. The silencing efficiency of shKIF18A in LX-2 cells was verified at the mRNA level by qPCR.** B** Silencing efficiency of shKIF18A was verified at the protein level by western blot. **C** Impact of KIF18A knockdown on cellular proliferation in the LX-2 cell line was assessed through CCK-8 assay. The findings demonstrate that decreased expression of KIF18A enhances cellular proliferative capacity.** D** EdU-incorporating live cells were detected by EdU proliferation assay. The cellular proliferative capacity was assessed by calculating the proportion of EdU-positive cells. The results revealed an enhanced cellular proliferative capacity following knockdown of KIF18A. **E** Flow cytometry analysis of cell apoptosis. The silencing of KIF18A led to a substantial reduction in the rate of cellular apoptosis. *P < 0.05, **P < 0.01, and ***P < 0.001

The downregulation of KIF18A expression in HSCs in liver fibrosis has been confirmed. Subsequently, we will explore the repercussions of aberrant KIF18A expression on the activation and functionality of HSCs in the liver. To obtain HSCs (LX-2) with stable knockdown or overexpression of KIF18A, lentivirus-mediated gene transfection was applied in vitro. qRT-PCR revealed significant inhibition of KIF18A expression in LX-2 cells (Fig. 2A). Western blot was used to detect the knockdown efficiency of shKIF18A. For subsequent experiments, we utilized shKIF18A#1 and shKIF18A#2 due to their superior knockdown efficiency (Fig. 2B). The CCK-8 assay is utilized for evaluating cellular proliferative ability and cell viability. The CCK8 assay indicated that the knockdown of KIF18A increased the proliferation and viability of LX-2 cells (Fig. 2C). EdU assays showed that KIF18A knockdown increased the ratio of EdU‐positive LX-2 cells. This observation further supports the stimulatory effect of KIF18A knockdown on the proliferation of LX-2 cells (Fig. 2D). Due to the close association between astrocyte activation and apoptosis, we investigated the impact of KIF18A knockdown on astrocyte apoptosis through flow cytometry analysis [27]. The analysis of apoptotic cells revealed a significant decrease in KIF18A-knockdown LX-2 cells, indicating that the downregulation of KIF18A leads to a reduction in apoptotic rate and promotes activation of LX-2 cells. (Fig. 2E). We replicated the above experiment using primary HSCs isolated from liver tissue. Our findings revealed that HSCs extracted from fibrotic liver tissue exhibited higher cellular vitality compared to those from normal liver tissue. In addition, knockdown of KIF18A in primary HSCs enhanced cellular vitality and proliferative capacity (Supplementary Fig. S1A, B). In line with the observations in LX-2 cells, silencing KIF18A in primary HSCs led to a comparable reduction in cellular apoptosis (Supplementary Fig. S1C).

### KIF18A overexpression inhibits cell proliferation and promotes apoptosis of HSCs

**Fig. 3:**
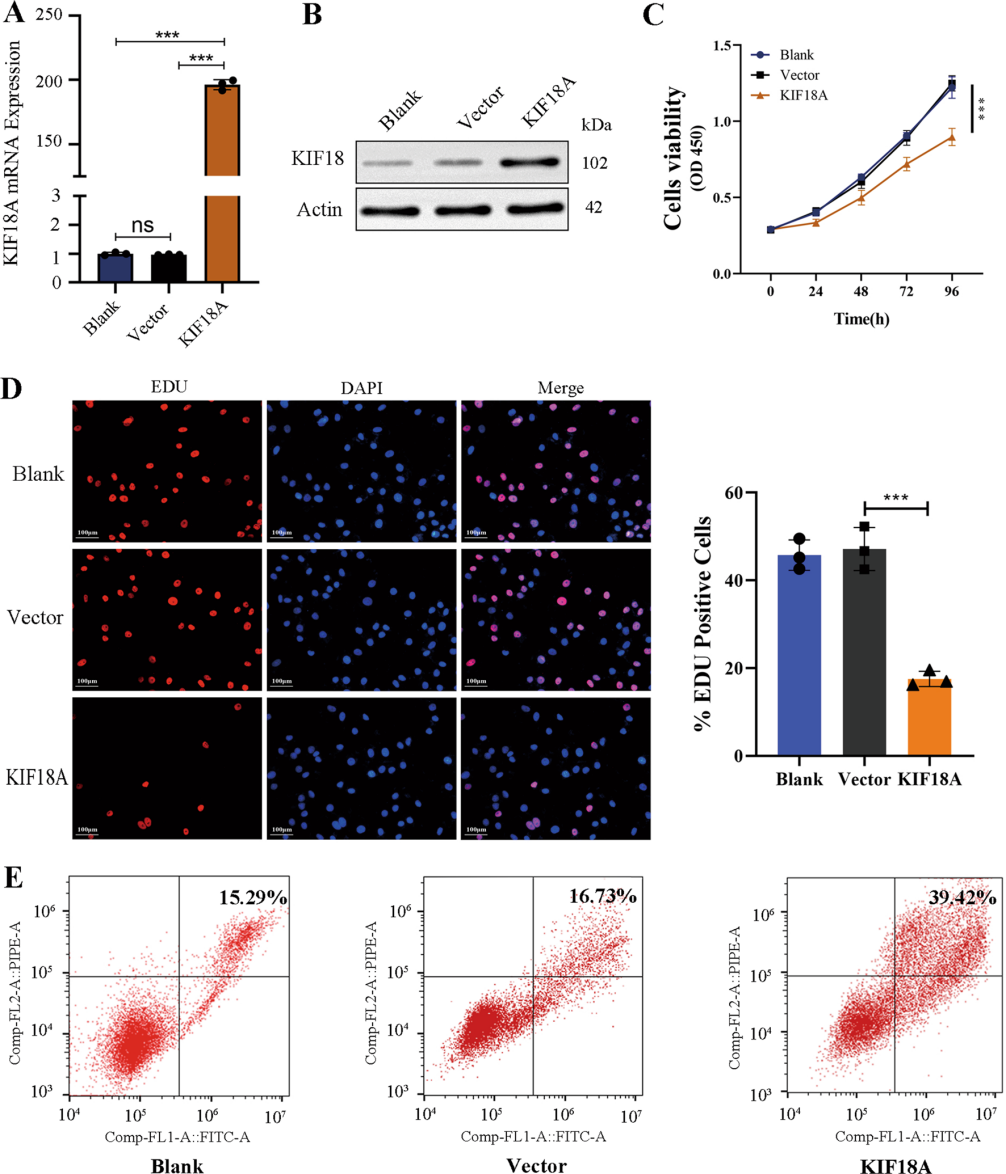
KIF18A overexpression inhibits cell proliferation and promotes apoptosis of HSCs. KIF18A was overexpressed using lentivirus and the effect of the overexpression of KIF18A on mRNA and protein expression levels in LX-2 cells was evaluated by RT-qPCR **(A)** and western blot analysis **(B)**. **C** KIF18A overexpression restrained the viability of LX-2 as detected by CCK8 assay. **D** EdU analysis showed that KIF18A overexpression suppressed the EdU-positive cells. **E** Overexpression of KIF18A was observed to increase LX2 cell apoptosis

To further investigate the impact of KIF18A overexpression on the functionality of HSCs, KIF18A was overexpressed by lentiviral transfection. The efficiency of KIF18A overexpression lentivirus in over-expressing KIF18A in LX-2 cells was determined by qRT-PCR and western blot. The qRT-PCR and western blot analyses indicated that lentiviral vector-mediated overexpression of KIF18A significantly elevated the levels of KIF18A mRNA and protein in LX-2 cells (Fig. 3A, B). KIF18A overexpression restrained the viability of LX-2 as detected by CCK8 assay (Fig. 3C). The EdU analysis revealed that overexpression of KIF18A enhanced the population of EdU-positive cells (Fig. 3D). In addition, the percentage of apoptotic cells was significantly increased in KIF18A-overexpressed cells (Fig. 3E). Upregulation of KIF18A in primary HSCs results in reduced cellular proliferation capacity and elevated apoptotic cell population (Supplementary Fig. S2A–C). Taken together, these findings suggest that the downregulation of KIF18A enhances cellular proliferation and suppresses apoptosis, whereas the upregulation of KIF18A shows the reverse effect.

### KIF18A induces liver fibrosis regression

**Fig. 4:**
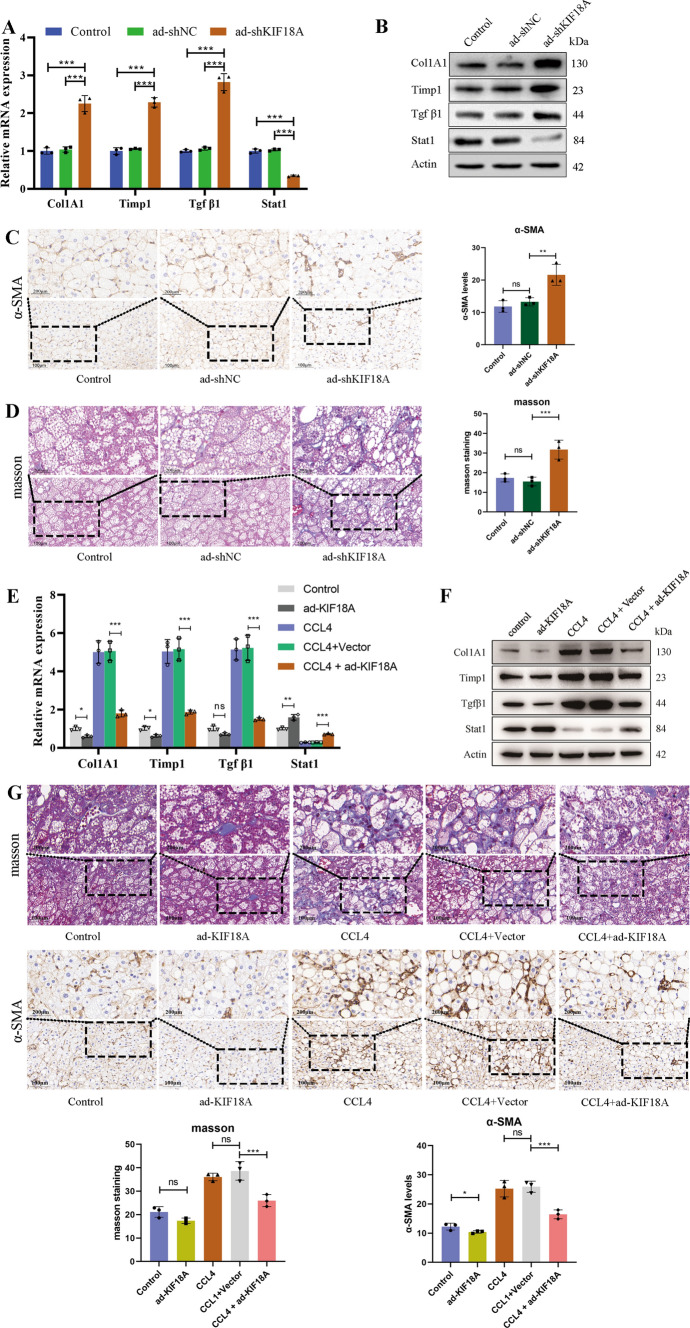
KIF18A induces liver fibrosis regression.** A** Performing tail vein injections of ad-shKIF18A to induce knockdown of KIF18A expression in the livers of three normal C57BL/6 mice. Three untreated mice and three mice injected with ad-shNC via the tail vein were designated as the control group. mRNA expression of fibrosis markers was assessed by RT-qPCR with KIF18A knockdown in mice. **B** Protein level of fibrosis markers was assessed by western blot with KIF18A knockdown in mice. **C** Masson’s trichrome staining and immunohistochemical staining for α-SMA can be utilized to evaluate the extent of liver fibrosis. Immunohistochemical staining for α-SMA reveals the initiation of liver fibrosis in mice after KIF18A knockdown. **D** Masson staining of the mouse liver in the KIF18A knockdown and control groups. **E** Established a liver fibrosis model in 9 C57BL/6 mice, evenly divided into 3 groups. The groups received treatments of no intervention, injection of empty adenovirus, and injection of adenovirus overexpressing KIF18A, respectively. Six mice were then evenly divided into two groups, serving as the control group and the KIF18A overexpression group. KIF18A overexpression decreased the mRNA levels of Col1A1, Timp1, and Tgfβ1 in the livers of both normal mice and those with CCL4-induced liver fibrosis, while concurrently upregulating Stat1 mRNA expression. **F** Protein level of fibrosis markers was assessed by western blot in different groups. The results suggest that the overexpression of KIF18A can attenuate the degree of liver fibrosis in mice. **G** Representative images of Masson trichrome staining and α-SMA immunostaining of mouse liver sections

After validating the effect of KIF18A on LX-2 in vitro, we next determined the roles of KIF18A in liver fibrosis initiation and progression in vivo by evaluating the expression of fibrosis markers. Col1A1, Timp1, and Tgfβ1, which are commonly regarded as fibrosis markers, were upregulated in liver fibrosis. In contrast, expression of the Signal transducer and activator of transcription 1 (Stat1) marker was downregulated in liver fibrosis. After KIF18A knockdown by injection of ad-shKIF18A in normal C57BL/6 mice, we found that the mRNA of Col1A1, Timp1, and Tgfβ1 increased. In contrast, the mRNA levels of Stat1 decreased (Fig. 4A). Meanwhile, the WB results exhibited significantly suppressed expression of Stat1 protein and elevated expression of Col1A1, Timp1, and Tgfβ1, consistent with the qRT-PCR results (Fig. 4B). We also evaluated liver fibrosis by Masson trichrome staining and immunohistochemistry detection of α-SMA protein. After KIF18A knockdown, the α-SMA protein levels significantly increased (Fig. 4C). Masson staining indicated that KIF18A knockdown promoted fibrosis progression (Fig. 4D). Overall, the above results suggested that KIF18A knockdown induced the development of liver fibrosis liver fibrosis in mice. In addition, we examined the influence of KIF18A overexpression on hepatic fibrosis in mice models. However, as the livers of healthy mice do not manifest fibrotic lesions, the alterations in fibrotic markers, including Col1A1, Timp1, and Tgfβ1, were not statistically significant after KIF18A overexpression. Therefore, we established a CCL4-induced mouse model of hepatic fibrosis and evaluated the effect of KIF18A overexpression on hepatic fibrosis in mice via intravenous injection of adenovirus. CCl4 increased the expression of Col1A1, Timp1, and Tgfβ1 but decreased that of Stat1. In contrast, Col1A1, Timp1, and Tgfβ1 decreased, and Stat1 increased in both mRNA and protein levels following KIF18A overexpression in liver fibrosis models (Fig. 4E, F). Masson staining and immunohistochemical images revealed that overexpression of KIF18A by ad-KIF18A injection significantly attenuated accumulated collagen and α-SMA levels induced by CCl4 (Fig. 4G). The present findings demonstrate that overexpression of KIF18A alleviates the severity of liver fibrosis in mice.

### KIF18A regulates the AKT/mTOR pathway and directly binds to TTC3

**Fig. 5:**
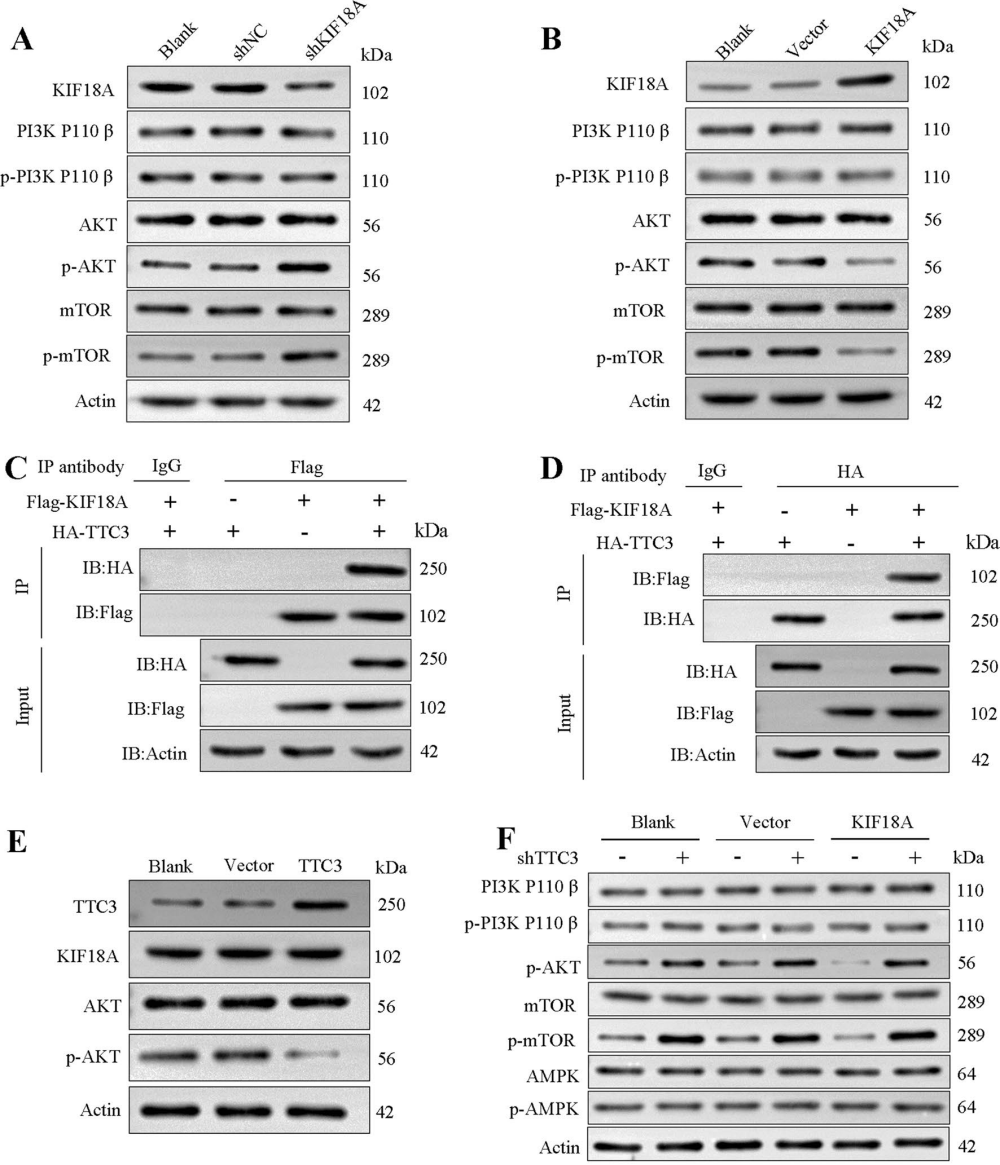
KIF18A regulates the AKT/mTOR pathway and directly binds to TTC3.** A** Induced knockdown of KIF18A in LX-2 cells and evaluated the protein expression and phosphorylation levels of the PI3K/AKT/mTOR-signaling pathway. **B** Following the overexpression of KIF18A in LX-2 cells, a decrease in the phosphorylation levels of p-AKT and p-mTOR was detected. However, no changes were observed in the protein expression and phosphorylation levels of various subunits of PI3K. **C** Co-transfected LX-2 cells with Flag-KIF18A and HA-TTC3 plasmids, followed by Co-IP experiments. Co‐IP showing Flag-KIF18A and HA-TTC3 interaction by using anti-Flag-KIF18A antibody. **D** Binding between Flag-KIF18A and HA-TTC3 was confirmed by Co-IP in LX-2 cells using anti-HA-TTC3 antibody. **E** Overexpression of TTC3 in LX-2 cells revealed downregulation of p-AKT levels. **F** p-AKT protein levels were inhibited after KIF18A overexpression and could be rescued by knockdown of TTC3. Adenosine monophosphate activated protein kinase (AMPK) is another important upstream molecule in the regulation of mTOR activity, and we found that TTC3 has no effect on the level of phosphorylation of AMPK

By examining multiple pathways related to liver fibrosis, such as TGFβ/Smad pathway, Ras/ERK pathway, JAK/STAT pathway, and PI3K/AKT pathway, we found overexpression or knockdown of KIF18A only affected the expression of the effector proteins of PI3K/AKT pathway. We constructed LX-2 cell line with stable knockdown of KIF18A and detected the protein expression levels of related signaling pathways in LX-2 cells after KIF18A knockdown. Western blotting showed p-AKT and p-mTOR expression increased after KIF18A knockdown in LX-2 cells (Fig. 5A). In contrast, p-AKT and p-mTOR decreased after KIF18A overexpression (Fig. 5B). We assessed the protein levels and phosphorylation levels of various subunits of PI3K. Interestingly, our findings revealed that the phosphorylation levels of PI3K subunits were not affected by KIF18A knockdown (Supplementary Fig. S3). These results indicate that KIF18A knockdown can activate the p-AKT/m-TOR-signaling pathway without influencing the phosphorylation levels of PI3K. To explore the molecular mechanism by which KIF18A regulates the AKT/mTOR pathway, IP and mass spectrometry (MS) were performed using anti-KIF18A antibody for the screening of proteins interactions with KIF18A (Supplementary Table S4). One of the proteins identified through MS, TTC3, has been reported to participate in the ubiquitination and degradation of p-AKT [28]. Therefore, we plan to conduct a series of experiments to confirm whether TTC3 is a crucial mediator in the regulatory impact of KIF18A on p-AKT. First, the direct binding of KIF18A and TTC3 in LX-2 cells was confirmed by a co-IP experiment (Fig. 5C, D). Then, western blotting assays indicated that TTC3 overexpression in LX-2 cells decreased the levels of p-AKT (Fig. 5E). The interaction between KIF18A and TTC3 has been experimentally confirmed, and TTC3 has been demonstrated to exert a negative regulatory effect on the protein levels of phosphorylated AKT. However, it remains unclear whether the regulation of p-AKT by KIF18A is mediated through TTC3. Hence, we conducted a rescue experiment. Elevated expression of KIF18A in LX-2 cells led to a reduction in p-AKT and p-mTOR levels. However, silencing of TTC3 counteracted the regulatory impact of KIF18A on p-AKT/p-mTOR (Fig. 5F). Based on the experimental findings, it has been demonstrated that TTC3 plays a mediating role in the regulatory effect of KIF18A on the p-AKT/p-mTOR-signaling pathway.

### KIF18A promotes p-AKT ubiquitination by enhancing binding between TTC3 and p-AKT

**Fig. 6:**
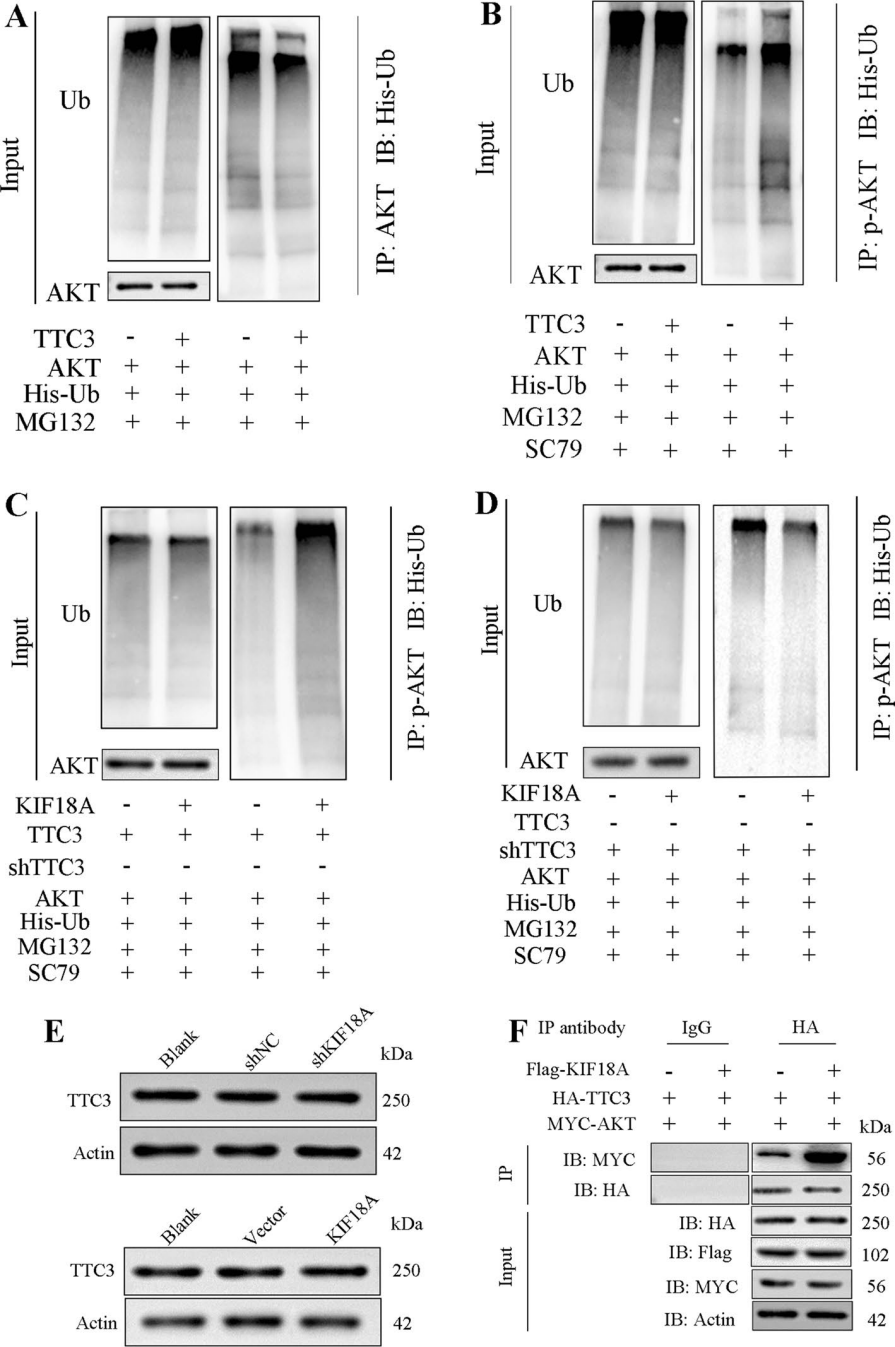
KIF18A promotes p-AKT ubiquitination by enhancing the binding of TTC3 and p-AKT.** A** In LX-2 cells co-transfected with AKT, TTC3, and ubiquitin plasmids, the ubiquitin-mediated modifications of AKT were examined. The results indicated that, compared to the control group, overexpression of TTC3 did not influence the ubiquitination levels of AKT. **B** Results of the ubiquitination assay indicated that the overexpression of TTC3 promoted the ubiquitination of p-AKT. **C** Detection of the ubiquitination levels of phosphorylated AKT (p-AKT) was performed after inducing overexpression of KIF18A to clarify the impact of KIF18A on the ubiquitination of p-AKT. Overexpression of KIF18A promoted the ubiquitination of p-AKT in LX-2 cells. **D** We conducted rescue experiments to verify whether the ubiquitination of p-AKT regulated by KIF18A is mediated by TTC3. The results indicated that the overexpression of KIF18A promoted the ubiquitination of p-AKT and can be reversed by TTC3 knockdown.** E** KIF18A overexpression or knockdown in LX-2 cells does not affect TTC3 protein levels.** F** Co-IP results illustrate that KIF18A promotes the interaction between TTC3 and AKT

In our previous experiments, we have demonstrated that KIF18A regulates the levels of p-AKT through TTC3. However, the mechanism by which TTC3 modulates p-AKT levels remains to be elucidated. Given the ubiquitin ligase activity of TTC3, our initial goal was to assess the potential of TTC3 in negatively regulating p-AKT by promoting protein ubiquitination and subsequent degradation. To evaluate the ubiquitination levels of AKT and p-AKT in LX-2 cells with KIF18A overexpression, a ubiquitin assay was performed. Results revealed that TTC3 could increase the ubiquitination levels of p-AKT rather than AKT (Fig. 6A, B). It is known that TTC3 functions as a ubiquitin ligase for active Akt. The ubiquitination and degradation of AKT by TTC3 may depend on the phosphorylation status of AKT. Then, we examined the impact of KIF18A on p-AKT ubiquitination and found that KIF18A overexpression could promote the ubiquitination level of p-AKT (Fig. 6C). To assess the role of KIF18A in promoting p-AKT ubiquitination mediated by TTC3, rescue experiments were performed using the LX-2 cell line. The results showed that TTC3 knockdown significantly blocked the promoting effects of KIF18A overexpression on p-AKT ubiquitination (Fig. 6D). To elucidate how KIF18A regulates p-AKT through TTC3, we investigated if KIF18A regulates TTC3 protein levels. The results showed that KIF18A overexpression or knockdown does not affect TTC3 protein levels (Fig. 6E). However, we found that KIF18A could enhance the interaction between TTC3 and p-AKT. Co-immunoprecipitation (Co-IP) results demonstrated an enhanced interaction between TTC3 and p-AKT in the presence of KIF18A overexpression (Fig. 6F). The above results illustrate that KIF18A promotes TTC3-mediated ubiquitination and degradation of p-AKT through enhancing protein interaction between TTC3 and p-AKT.

### YY1 is a transcription factor regulating KIF18A transcription

**Fig. 7:**
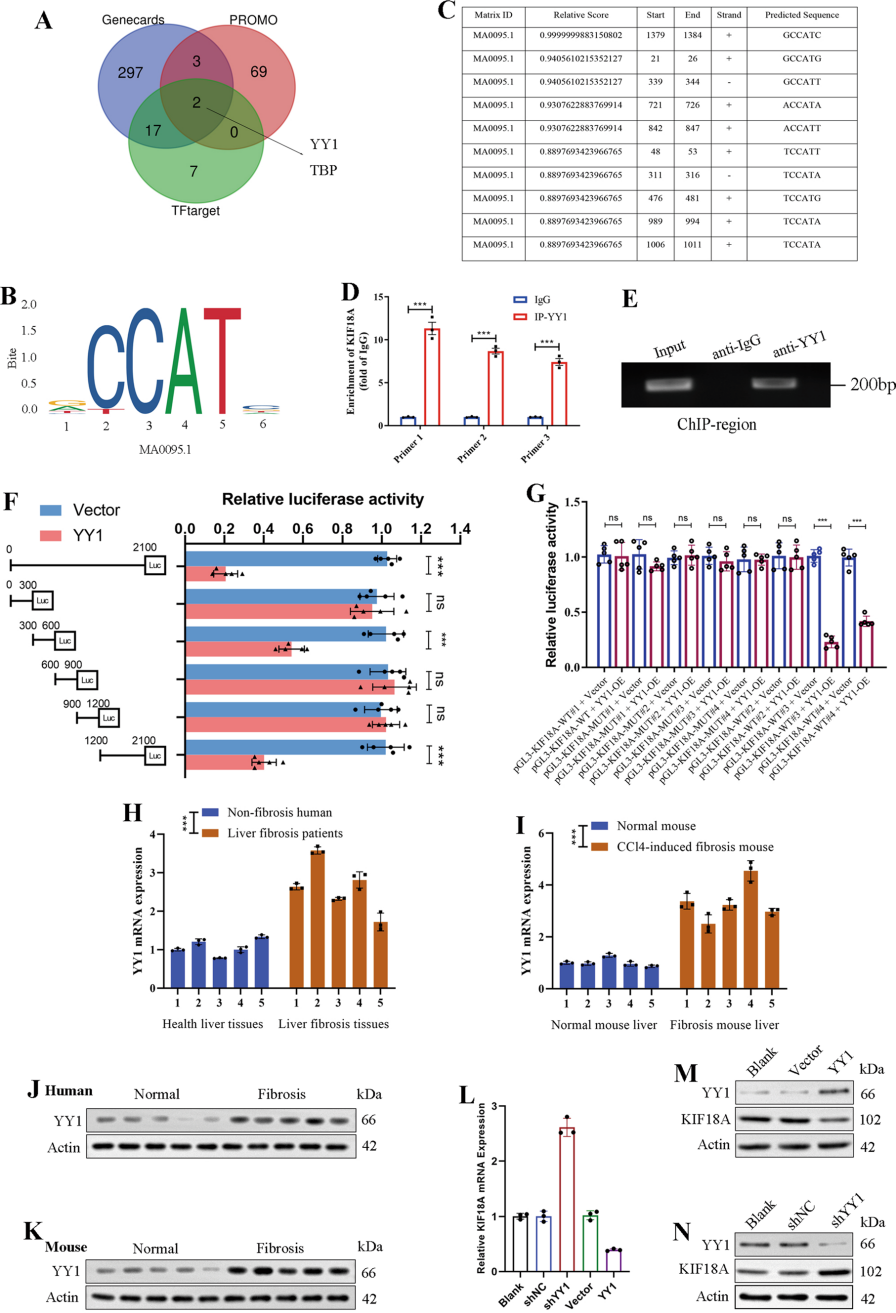
YY1 is a transcription factor regulating KIF18A transcription.** A** Predicted transcriptional factors of KIF18A using GeneCards, PROMO, and TFtarget databases. **B** According to the transcription factor prediction results, we found that YY1 recognizes the binding motif MA0095.1 within the promoter region of KIF18A. **C** Predicted binding site in the KIF18A promoter region where YY1 may bind. **D** To validate the interaction of YY1 with the promoter region of KIF18A, we conducted a ChIP experiment using anti-YY1 antibodies. PCR primers specific to the KIF18A promoter region were designed for PCR detection. The results demonstrated that YY1 can bind to the promoter region of KIF18A. **E** DNA electrophoresis gels show PCR products obtained after the reaction with ChIP-purified DNA. **F** Truncated plasmids containing distinct sequences of the KIF18A promoter region were constructed and co-transfected with YY1 overexpression plasmids into LX-2 cells. Subsequently, a dual-luciferase reporter gene assay was performed. The results suggest that YY1 may bind to the 300–600 bp and 1200–2100 bp regions of the KIF18A promoter. **G** We generated mutant plasmids and performed a dual-luciferase reporter gene assay. The findings revealed that YY1 directly interacts with the KIF18A promoter region at positions 476–481 bp and 1379–1384 bp. **H** mRNA expression level of YY1 in human normal liver tissue and fibrotic liver samples. **I** mRNA expression level of YY1 in mouse normal liver tissue and CCl4-induced mouse fibrotic liver samples. **J** Protein level of YY1 in human normal liver tissue and fibrotic liver samples. **K** Protein level of YY1 in mouse normal liver tissue and CCl4-induced mouse fibrotic liver samples. **L** After inducing overexpression or knockdown of YY1, the mRNA levels of KIF18A were quantified using qRT-PCR. The findings demonstrated that YY1 exerts a negative regulatory effect on the transcription of KIF18A. **M** Overexpression of YY1 leads to a decrease in the protein level of KIF18A. **N** KIF18A protein expression levels were upregulated after YY1 knockdown

To further investigate the underlying causes of downregulation of KIF18A mRNA expression in HSCs during fibrosis. We initially considered the possibility that the decrease in KIF18A mRNA expression is due to inhibition of DNA transcription. We defined the region 2000 bp upstream and 100 bp downstream of the transcription start site of KIF18A as the KIF18A transcription promoter region. We searched for transcription factors that bind to KIF18A promoter region to understand its transcriptional regulatory mechanism. Data from GeneCards, PROMO, and TFtarget databases, revealed that YY1 and TBP play significant roles in regulating KIF18A expression (Fig. 7A). YY1 was selected for the next stage of this study based on its superior binding capacity prediction scores. The transcription factor YY1 could recognize the motif MA0095.1 (Fig. 7B). The human KIF18A promoter region was analyzed for transcription factor binding sites, and the results revealed the presence of multiple YY1 recognition sites (Fig. 7C). The ChIP assay performed in LX-2 cells confirmed the interaction between the KIF18A promoter region and the YY1 promoter region (Fig. 7D, E). To precisely identify the binding sites between YY1 and the KIF18A promoter region, we generated truncated plasmids. The plasmid overexpressing YY1 was co-transfected with truncated plasmids harboring luciferase into LX-2 cells. The results of the dual luciferase assay demonstrated that YY1 inhibits the expression of luciferase in the truncated plasmids with sequence lengths of 300–600 bp and 1200–2100 bp (Fig. 7F). Due to the presence of three predicted binding sites within the 300–600 bp region of the promoter, we constructed four mutant plasmids for the 200–320 bp, 320–450 bp, 450–600 bp, and 1200–1350 bp regions (Supplementary Fig. S4). The dual luciferase reporter gene assay using the mutant plasmids revealed that the 476–481 bp and 1379–1384 bp regions within the YY1 promoter were confirmed to be binding sites for YY1 (Fig. 7G). Subsequently, we investigated the mRNA expression level of YY1 in human fibrotic liver tissues. We observed that it was significantly higher than in normal liver tissues (Fig. 7H). In addition, we observed upregulated YY1 mRNA expression in mouse fibrotic liver tissues compared to normal mouse liver tissues (Fig. 7I). Similarly, western blot analysis indicated that increased levels of KIF18A expression were detected in the fibrotic livers of both humans and mice (Fig. 7J, K). In addition, overexpression of YY1 decreased the mRNA expression of KIF18A, while the knockdown of YY1 upregulated its expression (Fig. 7L). We employed Western blot analysis to assess the KIF18A protein levels in LX-2 cells after YY1 knockdown or overexpression. The findings revealed a consistent correlation with changes in mRNA, suggesting that YY1 exerts a suppressive effect on KIF18A protein expression (Fig. 7M, N). In summary, our results demonstrated that YY1 could bind the KIF18A promoter region and inhibit KIF18A transcription.

### YY1 regulates p-AKT through KIF18A and TTC3

**Fig. 8:**
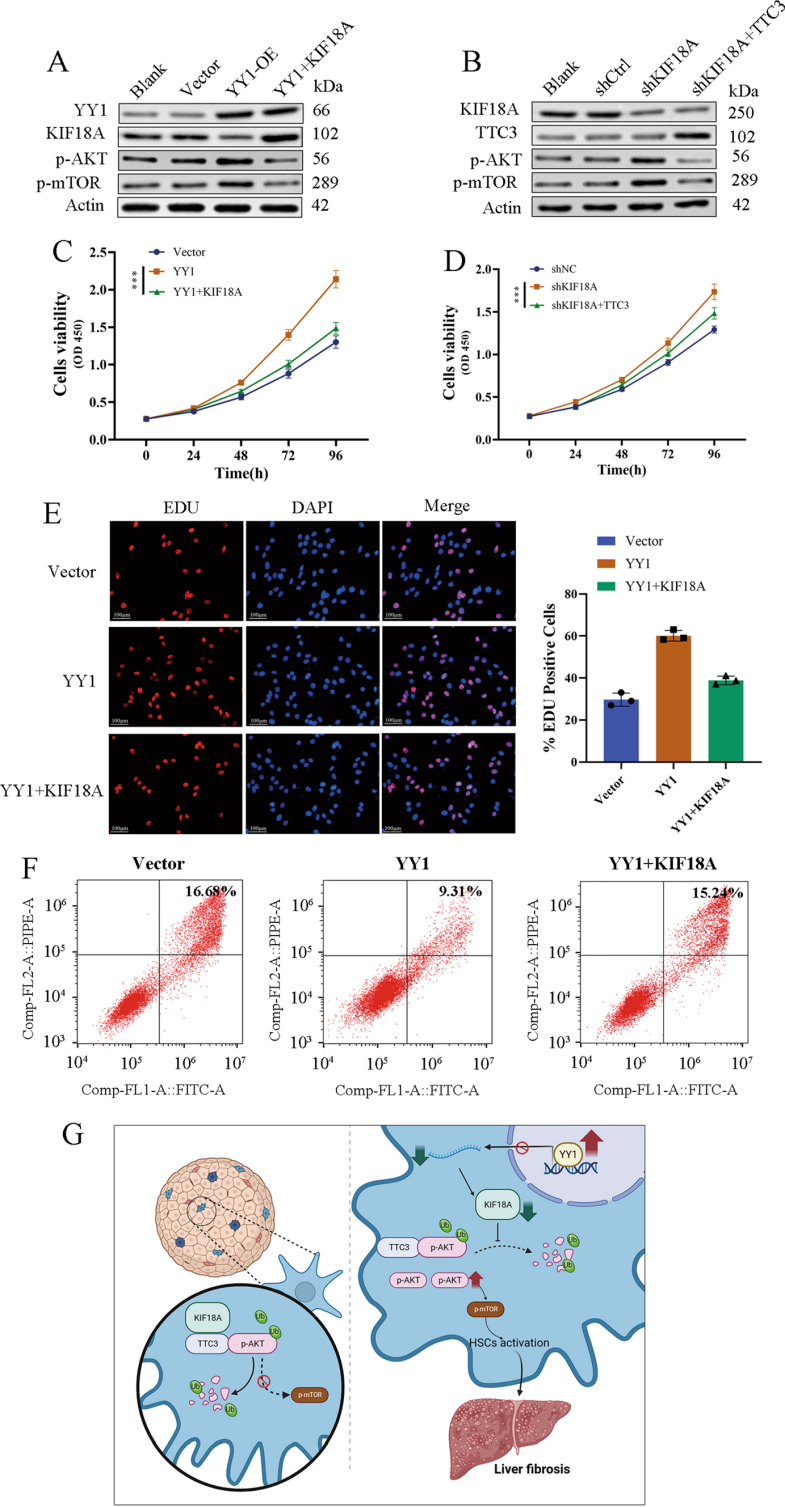
YY1 regulates p-AKT through KIF18A and TTC3.** A** YY1 overexpression induces the activation of the AKT/mTOR pathway in LX-2 cells, which was also reversed by KIF18A overexpression. **B** KIF18A knockdown induces the activation of the AKT/mTOR pathway, which was also reversed by TTC3 overexpression.** C** YY1 overexpression significantly promoted the proliferation of LX-2 determined by the CCK-8 assay, which was also reversed by KIF18A overexpression. **D** KIF18A knockdown promoted the proliferation of LX-2 cells, which was also reversed by TTC3 overexpression. **E** EdU assay showed more EdU-positive cells in the YY1 overexpressed group, which was also reversed by KIF18A overexpression. **F** Overexpression of YY1 decreased cell apoptosis, which was reversed by overexpression of KIF18A. **G** Mechanism diagram illustrates the mechanism by which the downregulation of KIF18A leads to increased levels of p-AKT, subsequently activating the AKT/mTOR-signaling pathway, and ultimately promoting the initiation and progression of liver fibrosis

To substantiate the regulatory mechanism of the YY1/KIF18A/TTC3-signaling axis, in vitro rescue experiments were performed. The overexpression of YY1 in LX-2 cells elevated the levels of p-AKT and p-mTOR, a process partially mitigated by the overexpression of KIF18A (Fig. 8A). The knockdown of KIF18A significantly increased the expression levels of p-AKT and p-mTOR, which could be reversed by the overexpression of TTC3 (Fig. 8B). The above findings demonstrate that YY1 modulates the p-AKT/p-mTOR-signaling pathway through KIF18A. KIF18A exerts negative regulation on the p-AKT/p-mTOR-signaling pathway by interacting with TTC3. The CCK8 assay showed that the proliferation ability of LX-2 cells was enhanced after YY1 overexpression. The cell proliferation phenotype was partially reversed after transfecting with the KIF18A‐overexpressing plasmid (Fig. 8C). Overexpression of TTC3 reverses the promotion of LX-2 cell proliferation induced by KIF18A knockdown (Fig. 8D). EdU assays revealed similar effects that overexpression of KIF18A in cells could significantly reverse the cell proliferation promoted by YY1 (Fig. 8E). The cell apoptosis assay demonstrated a decrease in the ratio of apoptotic cells with the overexpression of YY1, an effect that was partially reversed by the overexpression of KIF18A (Fig. 8F). A mechanism diagram was generated to visually depict the molecular regulatory mechanisms discussed in this paper. In the context of liver fibrosis, YY1 is aberrantly upregulated, resulting in the transcriptional suppression of KIF18A and its protein expression. KIF18A interacts with TTC3 and then facilitates the TTC3-mediated ubiquitination and degradation of p-AKT, leading to a reduction in p-AKT protein levels. The downregulation of KIF18A subsequently results in elevated p-AKT levels, ultimately activating the AKT/mTOR-signaling pathway and promoting the initiation and progression of liver fibrosis (Fig. 8G).

## Discussion

Hepatic fibrosis is a complication of chronic liver damage characterized by excessive extracellular matrix accumulation in liver tissue and associated with significant morbidity and mortality [29–31]. Currently, there is an urgent need to develop anti-liver fibrosis agents, as currently available therapies for liver fibrosis exhibit limited efficacy.

KIF18A, a motor protein kinesin family member, plays a crucial role in multiple critically involved cellular processes such as cell migration, cell division, cell shape, and cytoskeleton dynamics [32, 33]. KIF18A is overexpressed in several types of cancer, and the overexpression of KIF18A in colorectal cancer was significantly correlated with peritoneal dissemination [34]. Nevertheless, the role of KIF18A in benign liver diseases such as hepatic fibrosis is not well-established. In this study, we corroborated that KIF18A is expressed at low levels in both human and mice liver fibrosis tissues, indicating the potentially important role of KIF18A in the pathogenesis of liver fibrosis. These findings suggest that KIF18A could serve as a novel target for developing novel interventions against liver fibrosis.

To determine the mechanism of KIF18A, we explored the downstream-signaling pathway of KIF18A. We found that KIF18A could directly bind TTC3 and affect the expression of the effector proteins of the PI3K/AKT pathway. TTC3 is an Akt-specific E3 ligase that can interact preferentially with p-Akt and induce polyubiquitination and subsequent [28]. It is well-established that AKT/mTOR is an influential signal transduction pathway for cell survival and apoptosis [35]. Besides, the AKT/mTOR-signaling pathway can reportedly inhibit autophagy, which is important in regulating liver pathophysiology [36]. In conclusion, these results suggest that KIF18A could bind to TTC3, regulating the AKT/mTOR-signaling pathway by promoting p-AKT ubiquitination and degradation and eventually deactivating hepatic stellate cells.

It is now understood that YY1 plays multiple roles in development, cellular proliferation and apoptosis [37]. Besides, it plays dual roles as an activator or repressor of gene transcription [38]. In this study, YY1 was identified as the upstream regulator of KIF18A. Riquet et al. found that YY1 promotes liver fibrosis by binding to the COL1A1 proximal promoter and functioning as the positive regulator of constitutive activity in fibroblasts [39]. In the present study, we observed that YY1 acts as a transcription repressor to inhibit the expression of KIF18A, and uncovered a hitherto undocumented mechanism by which YY1 regulates liver fibrosis in HSCs.

Despite these promising findings, there are still some unresolved issues. Indeed, further studies are necessary to determine the efficacy of targeting KIF18A for treating liver fibrosis. One potential approach could involve developing AAV vectors that express the KIF18A gene, as they have been documented to be safe and effective for clinical use. Alternatively, small molecule activator drugs that target KIF18A could be developed in the future. Besides, the underlying mechanisms of how KIF18A impacts the protein binding of TTC3 and p-AKT warrant further investigation, despite our finding that KIF18A can promote TTC3 binding to p-AKT.

In summary, our study sheds light on the YY1/KIF18A/TTC3-signaling pathway and its effects on AKT/TOR, providing a basis for future research into liver fibrosis treatment.

### Supplementary Information

Below is the link to the electronic supplementary material.

Supplementary file1 (TIF 2169 KB)

Supplementary file2 (TIF 1979 KB)

Supplementary file3 (TIF 2571 KB)

Supplementary file4 (TIF 1468 KB)

Supplementary file5 (PDF 38 KB)

Supplementary file6 (PDF 58 KB)

Supplementary file7 (PDF 38 KB)

Supplementary file8 (PDF 28 KB)

## Data Availability

All data in this article are publicly available. All data generated in this study are available from the corresponding author upon reasonable request.
