# Supplementary figures and images for: KIF18A inactivates hepatic stellate cells and alleviates liver fibrosis through the TTC3/Akt/mTOR pathway
Source: Cell Mol Life Sci. 2024 Feb 19;81(1):96. doi: 10.1007/s00018-024-05114-5 (PMC10876760; doi:10.1007/s00018-024-05114-5)

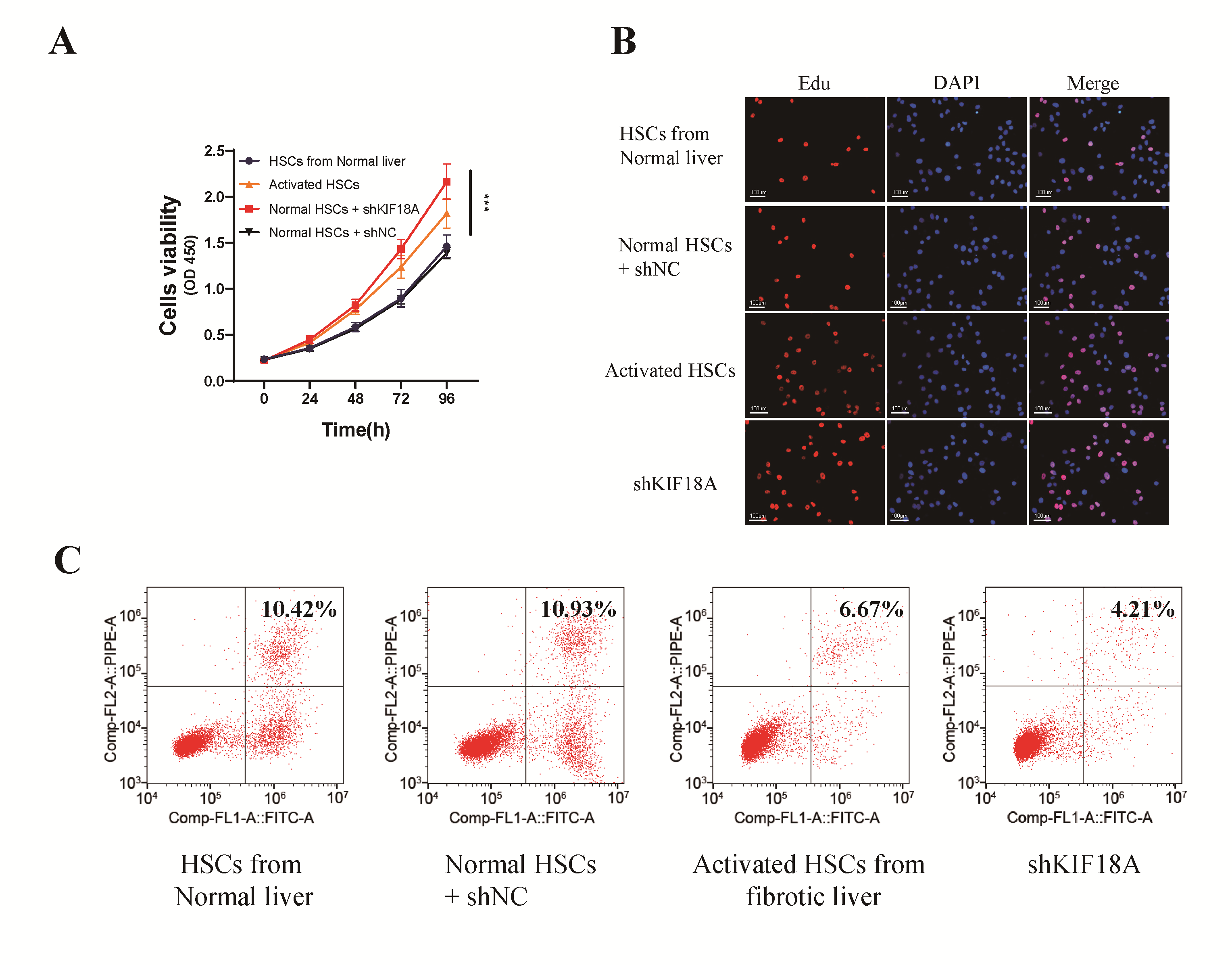

Supplement: Supplementary file 1 — Supplementary file1 (TIF 2169 KB) [file 18_2024_5114_MOESM1_ESM.tif]

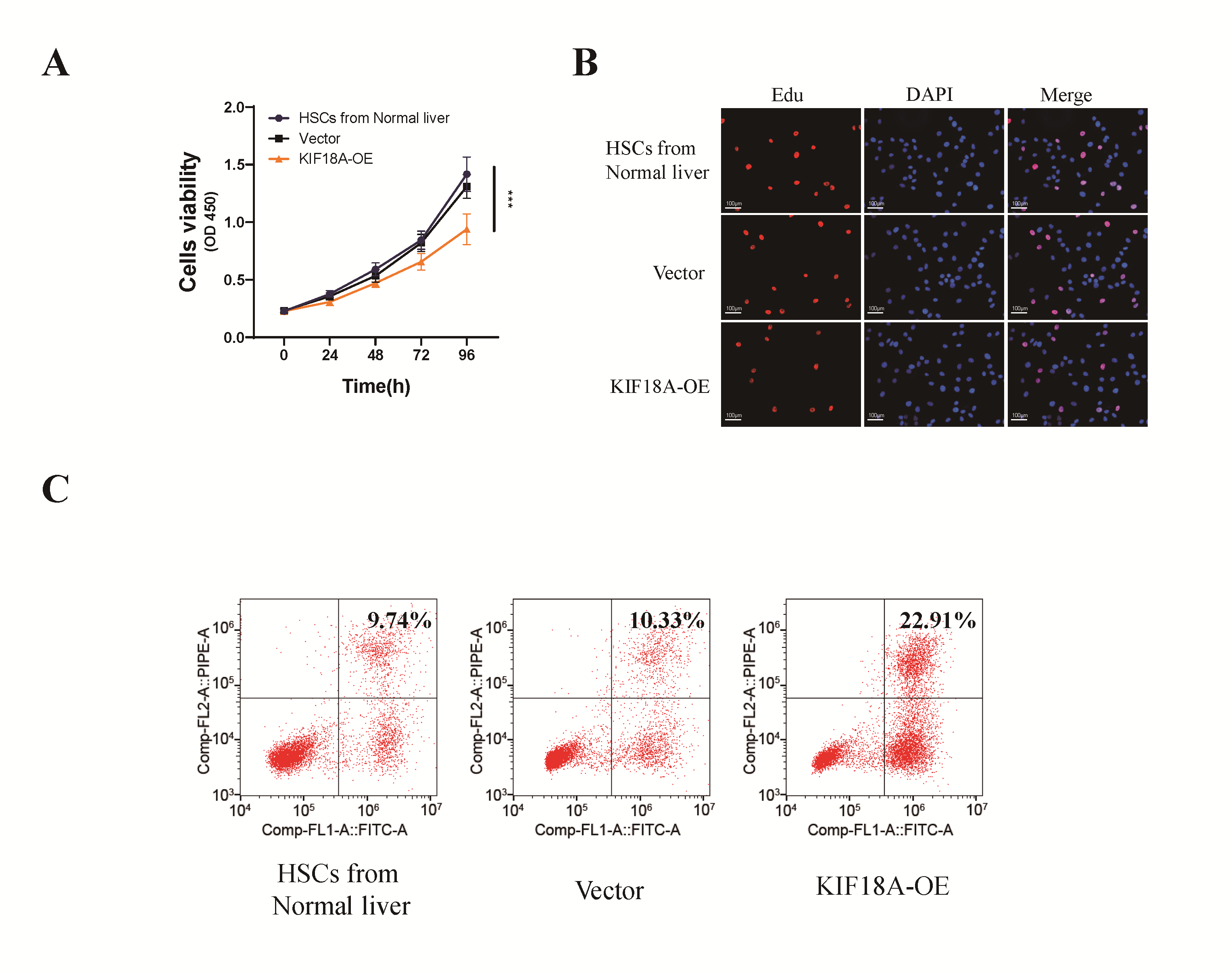

Supplement: Supplementary file 2 — Supplementary file2 (TIF 1979 KB) [file 18_2024_5114_MOESM2_ESM.tif]

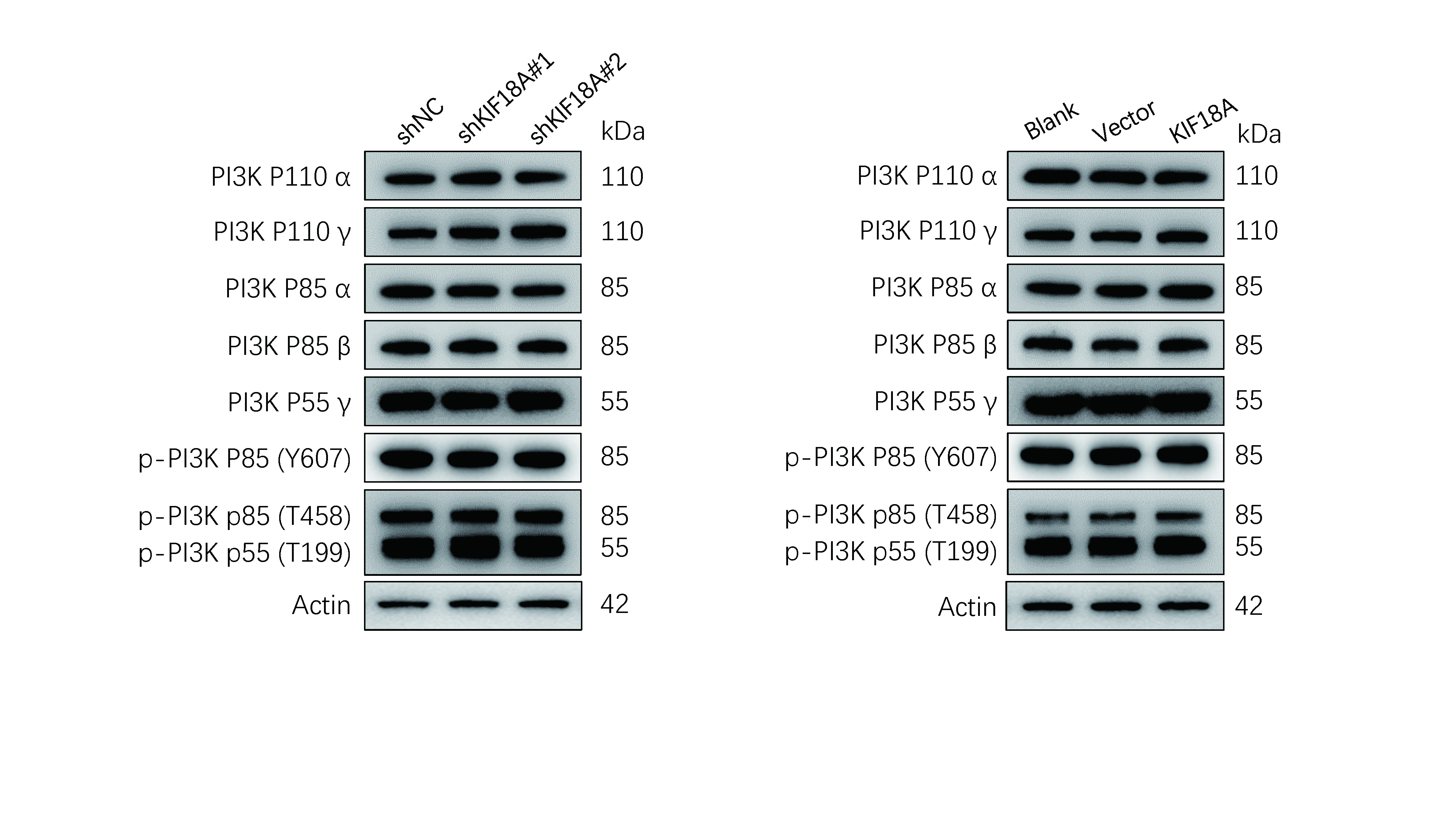

Supplement: Supplementary file 3 — Supplementary file3 (TIF 2571 KB) [file 18_2024_5114_MOESM3_ESM.tif]

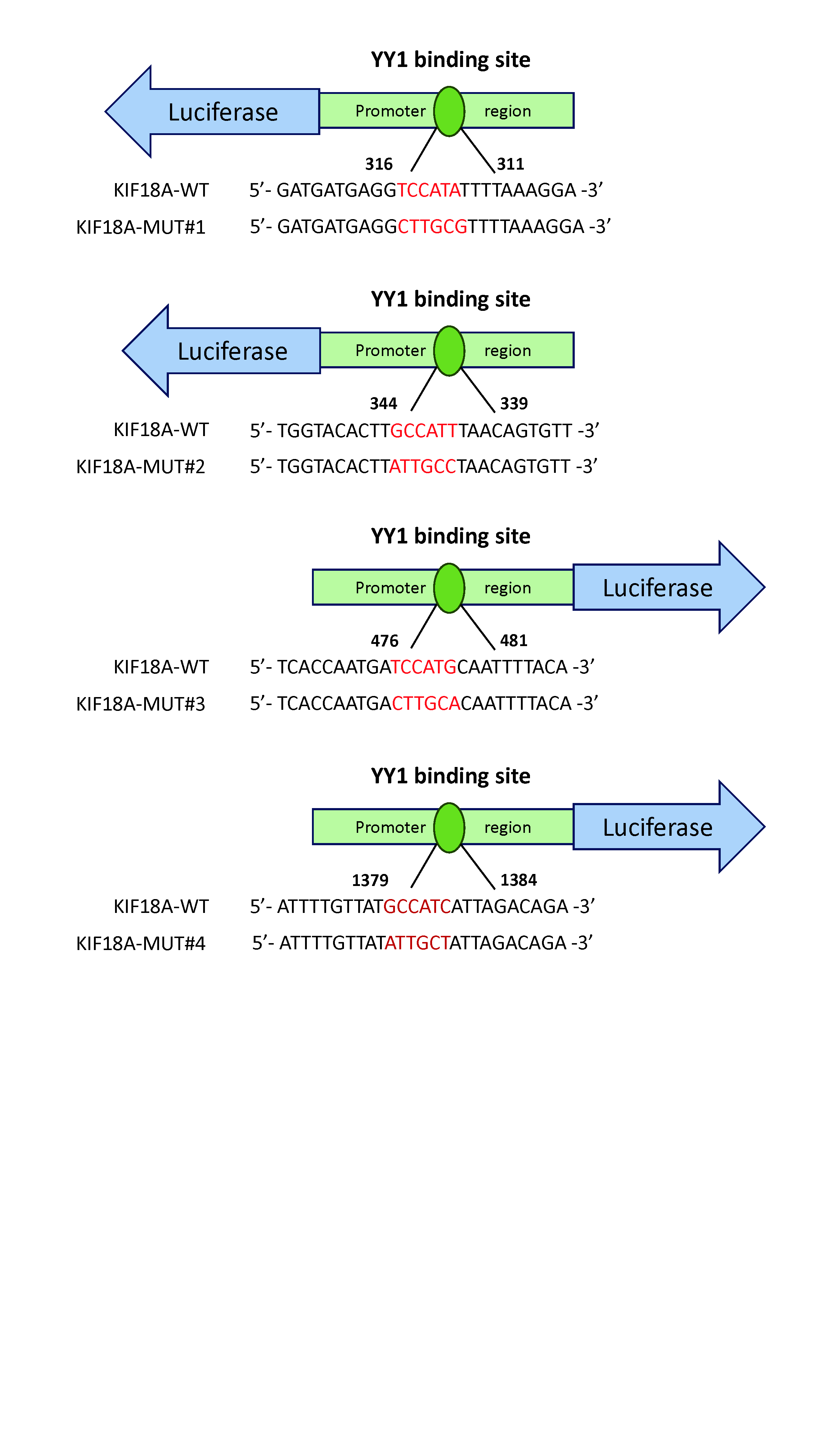

Supplement: Supplementary file 4 — Supplementary file4 (TIF 1468 KB) [file 18_2024_5114_MOESM4_ESM.tif]
