# Supplementary material for: KIF18A inactivates hepatic stellate cells and alleviates liver fibrosis through the TTC3/Akt/mTOR pathway
Source: Cell Mol Life Sci. 2024 Feb 19;81(1):96. doi: 10.1007/s00018-024-05114-5 (PMC10876760; doi:10.1007/s00018-024-05114-5)
Supplement: Supplementary file 5 — Supplementary file5 (PDF 38 KB) [file 18_2024_5114_MOESM5_ESM.pdf]

Species	Gene Name	Forward primer
Mouse	KIF18A	ATCTGGGAAAACTCACACAATGC
Mouse	β-actin	GGCTGTATTCCCCTCCATCG
Mouse	STAT1	TCACAGTGGTTCGAGCTTCAG
Mouse	Col1A1	GCTCCTCTTAGGGGCCACT
Mouse	Timp1	CGAGACCACCTTATACCAGCG
Mouse	Tgfβ1	CCACCTGCAAGACCATCGAC
Mouse	YY1	GTGGTTGAAGAGCAGATCATTGG
Human	YY1	ACGGCTTCGAGGATCAGATTC

Reverse primer
GGCAGTGCTACACTCTTTCTCT
CCAGTTGGTAACAATGCCATGT
CGAGACATCATAGGCAGCGTG
ATTGGGGACCCTTAGGCCAT
ATGACTGGGGTGTAGGCGTA
CTGGCGAGCCTTAGTTTGGAC
TTGCTTAGGGTCTGAGAGGTC
TGACCAGCGTTTGTTCAATGT
