# Supplementary material for: KIF18A inactivates hepatic stellate cells and alleviates liver fibrosis through the TTC3/Akt/mTOR pathway
Source: Cell Mol Life Sci. 2024 Feb 19;81(1):96. doi: 10.1007/s00018-024-05114-5 (PMC10876760; doi:10.1007/s00018-024-05114-5)
Supplement: Supplementary file 6 — Supplementary file6 (PDF 58 KB) [file 18_2024_5114_MOESM6_ESM.pdf]

Antibody name	Cat No.	Product no.	Ratio of concentration
PI 3 Kinase p85 beta	ab313427	abcam	1:1000
phospho- PI 3 Kinase p85 alpha (phospho Y607)	ab182651	abcam	1:500
Phospho-Akt (Ser473) (D9E) XP® Rabbit mAb #4060	#4060	cell signaling Technology	20μg for IP;1:2000 for WB
PI3 Kinase p110 gamma (D55D5) Rabbit mAb	#5405	cell signaling Technology	1:1000
PI3 Kinase p110 alpha (C73F8) Rabbit mAb	#4249	cell signaling Technology	1:1000
Phospho-PI3 Kinase p85 (Tyr458)/p55 (Tyr199)	#17366	cell signaling Technology	1:1000
COL1A1 (E8F4L)	#72026	cell signaling Technology	1:1000
TIMP1 (E1Y1U)	#63363	cell signaling Technology	1:1000
PI3 Kinase p85 Alpha Monoclonal antibody	60225-1-Ig	Proteintech	1:2000
PI3 Kinase p110 Beta Polyclonal antibody	20584-1-AP	Proteintech	1:1000
KIF18A Polyclonal antibody	19245-1-AP	Proteintech	2μg for IP;1:2000 for WB
Beta Actin Polyclonal antibody	20536-1-AP	Proteintech	1:2000
STAT1 Polyclonal antibody	10144-2-AP	Proteintech	1:5000
mTOR Monoclonal antibody	66888-1-Ig	Proteintech	1:5000
Phospho-mTOR (Ser2448) Monoclonal antibody	67778-1-Ig	Proteintech	1:3000
YY1 Monoclonal antibody	66281-1-Ig	Proteintech	2μg for ChIP; 1:10000 for WB
HA tag Polyclonal antibody	51064-2-AP	Proteintech	2μg for ChIP; 1:2000 for WB
Flag tag Monoclonal antibody	66008-4-Ig	Proteintech	2μg for ChIP; 1:10000 for WB
His-Tag Monoclonal antibody	66005-1-Ig	Proteintech	2μg for ChIP; 1:10000 for WB
Mouse IgG	B900620	Proteintech	2μg for ChIP
Rabbit IgG	B900610	Proteintech	2μg for ChIP
PI3K p55 gamma Antibody	MA5-31995	ThermoFisher	1:500

PIK3R2 (P85 beta)Polyclonal Antibody	PA5-84807	ThermoFisher	1:3000
phospho-PI3 Kinase p110 beta(Ser1070)	BS-6417R	ThermoFisher	1:200
