# Supplementary material for: KIF18A inactivates hepatic stellate cells and alleviates liver fibrosis through the TTC3/Akt/mTOR pathway
Source: Cell Mol Life Sci. 2024 Feb 19;81(1):96. doi: 10.1007/s00018-024-05114-5 (PMC10876760; doi:10.1007/s00018-024-05114-5)
Supplement: Supplementary file 7 — Supplementary file7 (PDF 38 KB) [file 18_2024_5114_MOESM7_ESM.pdf]

Species	Gene Name	Forward primer	Reverse primer
Human	Primer 1	TCTTCACCAATGATCCATGCAA	TCAGAATTGAGTCATTTGTCTCTGA
Human	Primer 2	ATTACCTACGTCTCATGGGCTG	ATTTGTGTGCTGCTGGTCTTG
Human	Primer 3	CGTATTTCCGATTTGTTCCAGGT	ACATTCTCTGTCTAATGATGGCA
