# Supplementary material for: KIF18A inactivates hepatic stellate cells and alleviates liver fibrosis through the TTC3/Akt/mTOR pathway
Source: Cell Mol Life Sci. 2024 Feb 19;81(1):96. doi: 10.1007/s00018-024-05114-5 (PMC10876760; doi:10.1007/s00018-024-05114-5)
Supplement: Supplementary file 8 — Supplementary file8 (PDF 28 KB) [file 18_2024_5114_MOESM8_ESM.pdf]

Accession	Gene	Protein	Description	Mw(kDa)	Length	Peptides
Q96EK5	KBP	KIF-binding protein	KIF-binding protein OS=Homo sapiens OX=9606 GN=KBP PE=1 SV=3	71.814	621	10
Q9UJX5	APC4	Anaphase-promoting complex	Anaphase-promoting complex subunit 4 OS=Homo	92.116	808	7
F8W881	FAM120C	Constitutive coactivator of PPAR-gamma-like protein 2	Constitutive coactivator of PPAR-gamma-like protein 2 OS=Homo sapiens OX=9606 GN=FAM120C PE=1 SV=2	99.352	895	6
P62136	PP1A	Serine/threonine-protein	Serine/threonine-protein phosphatase PP1-alpha	37.512	330	5
Q13042	CDC16	Cell division cycle protein 16 homolog	Cell division cycle protein 16 homolog OS=Homo sapiens OX=9606 GN=CDC16 PE=1 SV=1	71.656	620	5
P53804	TTC3	E3 ubiquitin-protein ligase TTC3	E3 ubiquitin-protein ligase TTC3 OS=Homo sapiens	229.869	2025	5
Q9H2P0	ADNP	Activity-dependent neuroprotector	Activity-dependent neuroprotector homeobox protein	123.563	1102	5
Q01082	SPTBN1	Spectrin beta chain, non-erythrocytic 1	Spectrin beta chain, non-erythrocytic 1 OS=Homo sapiens OX=9606 GN=SPTBN1 PE=1 SV=2	274.609	2364	4
P62807	H2B1C	Histone H2B type 1-C/E/F/G/I	Histone H2B type 1-C/E/F/G/I OS=Homo sapiens OX=9606 GN=H2B1C PE=1 SV=1	13.906	126	3
B2RXH8	HNRC2	Heterogeneous nuclear ribonucleoprotein C-like 2	Heterogeneous nuclear ribonucleoprotein C-like 2 OS=Homo sapiens OX=9606 GN=HNRC2 PE=3 SV=1	32.072	293	3
Q9UMF0	ICAM5	Intercellular adhesion molecule 5	Intercellular adhesion molecule 5 OS=Homo sapiens	97.116	924	3
Q8WUP2	FBLI1	Filamin-binding LIM protein 1	Filamin-binding LIM protein 1 OS=Homo sapiens OX=9606 GN=FBLI1 PE=1 SV=1	40.67	373	3
P29590	PML	Protein PML	Protein PML OS=Homo sapiens OX=9606 GN=PML	97.551	882	2
P46013	KI67	Proliferation marker protein Ki-67	Proliferation marker protein Ki-67 OS=Homo sapiens	358.694	3256	2
Q12834	CDC20	Cell division cycle protein 20 homolog	Cell division cycle protein 20 homolog OS=Homo sapiens OX=9606 GN=CDC20 PE=1 SV=3	54.723	499	2
Q8NFT6	DBF4B	Protein DBF4 homolog B	Protein DBF4 homolog B OS=Homo sapiens OX=9606	67.243	615	2
Q96AY2	EME1	Crossover junction endonuclease EME1	Crossover junction endonuclease EME1 OS=Homo sapiens OX=9606 GN=EME1 PE=1 SV=3	63.252	570	2
Q15653	IKBB	NF-kappa-B inhibitor beta	NF-kappa-B inhibitor beta OS=Homo sapiens OX=9606	37.771	356	2
Q16821	PPR3A	Protein phosphatase 1 regulatory subunit 3A	Protein phosphatase 1 regulatory subunit 3A OS=Homo sapiens OX=9606 GN=PPR3A PE=1 SV=1	125.767	1122	2
P25788	PSA3	Proteasome subunit alpha type-3	Proteasome subunit alpha type-3 OS=Homo sapiens	28.433	255	2
Q9NYU2	UGGG1	UDP-glucose:glycoprotein	UDP-glucose:glycoprotein glucosyltransferase 1	177.19	1555	2
Q9H444	CHM4B	Charged multivesicular body protein 4b	Charged multivesicular body protein 4b OS=Homo sapiens OX=9606 GN=CHM4B PE=1 SV=2	24.95	224	2

Q8NI36	WDR36	WD repeat-containing protein 36	WD repeat-containing protein 36 OS=Homo sapiens	105.322	951	2
P25787	PSMA2	Proteasome subunit alpha type-2	Proteasome subunit alpha type-2 OS=Homo sapiens OX=9606 GN=TUBA1C PE=1 SV=1	25.899	234	2
P52597	HNRNPF	Heterogeneous nuclear	Heterogeneous nuclear ribonucleoprotein F OS=Homo	45.672	415	2
P46776	RPL27A	Large ribosomal subunit protein uL15	60S ribosomal protein L27a OS=Homo sapiens OX=9606 GN=RPL27A PE=1 SV=1	16.561	148	2
P14618	PKM	pyruvate kinase M1/2	pyruvate kinase M1/2 OS=Homo sapiens OX=9606	57.937	531	1
Q15185	PTGES3	Prostaglandin E synthase 3	Prostaglandin E synthase 3 OS=Homo sapiens OX=9606 GN=PTGES3 PE=1 SV=1	18.697	160	1
Q9UJX2	CDC23	Cell division cycle protein 23	Cell division cycle protein 23 homolog OS=Homo	68.834	597	1
P55060	XPO2	Exportin-2	Exportin-2 OS=Homo sapiens OX=9606 GN=XPO2 PE=1 SV=2	110.417	971	1
P49720	PSB3	Proteasome subunit beta type-3	Proteasome subunit beta type-3 OS=Homo sapiens	22.949	205	1
P0DO92	CDIPTOSP	CDIP transferase opposite strand, pseudogene	Putative protein T-ENOL OS=Homo sapiens OX=9606 GN=CDIPTOSP PE=4 SV=1	9.002	83	1
Q8IY92	SLX4	SLX4 structure-specific	SLX4 structure-specific endonuclease subunit	200.012	1834	1
Q8TDY2	RB1CC1	RB1-inducible coiled-coil protein 1	RB1-inducible coiled-coil protein 1 OS=Homo sapiens OX=9606 GN=RB1CC1 PE=1 SV=1	183.091	1594	1
Q9UL42	PNMA2	Paraneoplastic antigen Ma2	PNMA family member 2 OS=Homo sapiens OX=9606	41.509	364	1
P30519	HMOX2	Heme oxygenase 2	Heme oxygenase 2 OS=Homo sapiens OX=9606 GN=HMOX2 PE=1 SV=3	36.033	316	1
Q86UK0	ABCA12	Glucosylceramide transporter	ATP-binding cassette sub-family A member 12	293.237	2595	1
O15178	TBXT	T-box transcription factor T	T-box transcription factor T OS=Homo sapiens OX=9606 GN=TBXT PE=1 SV=3	47.443	435	1
P38398	BRCA1	Breast cancer type 1 susceptibility	Breast cancer type 1 susceptibility protein OS=Homo	207.721	1863	1
P62244	RPS15A	Small ribosomal subunit protein uS8	Small ribosomal subunit protein uS8 OS=Homo sapiens OX=9606 GN=RPS15A PE=1 SV=2	14.840	130	1
O75376	NCOR1	Nuclear receptor corepressor 1	Nuclear receptor corepressor 1 OS=Homo sapiens	270.210	2440	1
Q32NC0	CR021	UPF0711 protein C18orf21	UPF0711 protein C18orf21 OS=Homo sapiens	24.827	220	1
P36873	PP1G	Serine/threonine-protein phosphatase PP1-gamma catalytic subunit	Serine/threonine-protein phosphatase PP1-gamma catalytic subunit OS=Homo sapiens OX=9606 GN=PP1G PE=1 SV=1	36.984	323	1
Q5VSY0	GKAP1	G kinase-anchoring protein 1	G kinase-anchoring protein 1 OS=Homo sapiens OX=9606 GN=GKAP1 PE=2 SV=1	42.078	366	1
P28066	PSA5	Proteasome subunit alpha type-5	Proteasome subunit alpha type-5 OS=Homo sapiens	26.411	241	1

P25815	S100P	Protein S100-P	Protein S100-P OS=Homo sapiens OX=9606 GN=S100P PE=1 SV=1	10.400	95	1
P30260	CDC27	Cell division cycle protein 27	Cell division cycle protein 27 homolog OS=Homo	91.867	824	1
P06748	NPM1	Nucleophosmin-1	nucleophosmin 1 OS=Homo sapiens OX=9606 GN=NPM1 PE=1 SV=2	32.575	294	1
